# Effects of a Functional Ice Cream Enriched with Milk Proteins on Bone Metabolism: A Feasibility Clinical Study and In Vitro Investigation

**DOI:** 10.3390/nu15020344

**Published:** 2023-01-10

**Authors:** Samantha Maurotti, Yvelise Ferro, Roberta Pujia, Miriam Frosina, Angela Sciacqua, Rosario Mare, Elisa Mazza, Nadia Geirola, Stefano Romeo, Arturo Pujia, Tiziana Montalcini

**Affiliations:** 1 Department of Clinical and Experimental Medicine, University “Magna Græcia” of Catanzaro, 88100 Catanzaro, Italy; 2 Department of Medical and Surgical Sciences, University “Magna Græcia” of Catanzaro, 88100 Catanzaro, Italy; 3 Sahlgrenska Center for Cardiovascular and Metabolic Research, Department of Molecular and Clinical Medicine, University of Gothenburg, 40530 Gothenburg, Sweden; 4 Research Center for the Prevention and Treatment of Metabolic Diseases, University “Magna Græcia” of Catanzaro, 88100 Catanzaro, Italy

**Keywords:** bone metabolism, milk proteins, bone mineral density, whey proteins, functional food

## Abstract

*Background*: Milk proteins (MPs) and their derivative whey proteins (WPs) are important components of human diet that might prevent bone loss. We aimed to investigate the effects of MP on the bones of postmenopausal women, along with the effects of WP on osteoblast cells. *Methods:* We conducted a feasibility controlled clinical study with 62 postmenopausal women who were asked to consume an MP-enriched ice cream. We also investigated the effect of WP on the ERK1/2 and AKT pathways, RUNX2, alkaline phosphatase, RANKL/OPG ratio, and COL1A of Saos-2. *Results*: After 12 weeks, we found a greater bone mineral density and bone alkaline phosphatase reduction in women who consumed the MP-enriched ice cream compared to the control group (*p* = 0.03 and *p* = 0.02, respectively). In Saos-2 cells, WP upregulated ERK1/2 and AKT pathways (*p* = 0.002 and *p* = 0.016), cell proliferation (*p* = 0.03), and osteoblast differentiation markers, along with downregulating RANKL/OPG (*p* < 0.001). Moreover, the inhibition of ERK1/2 by PD184253 reverted the effects on both the RUNX2 and ALP mRNA expression and cells proliferation (*p* = 0.028, *p* = 0.004, and *p* = 0.003, respectively) when treated with WP. *Conclusions*: WP upregulates cell proliferation, RUNX2, and alkaline phosphatase through the activation of the ERK1/2 pathways on Saos-2. These mechanisms probably contribute to preventing bone loss in postmenopausal women.

## 1. Introduction

Osteoporosis (OP) is a common chronic metabolic disease of the skeleton, characterized by a low bone mineral density (BMD) and micro-architectural deterioration of bone tissue, which leads to an increased bone fragility and fracture risk [1].

In the United States, there are approximately 10 million individuals aged over 50 who are affected by OP, with a similar burden of disease observed in the United Kingdom [2]. For example, in the U.K., women older than 50 have a four-fold higher rate of OP and a two-fold higher rate of osteopenia than men [3]. The risk of OP-related fractures in women is approximately 40% [4]. The economic burden of OP fractures every year is approximately USD 17.9 billion in the United States and GBP 4 billion in the United Kingdom [2], thus representing a major global health problem. Several effective and safe pharmacological agents are available to reduce the risk of OP fractures [5], but often the individuals do not start OP treatment or they underestimate the issue, causing an increase in the clinical and economic burden [6]. Many determinants of patients’ non-adherence to anti-osteoporotic therapy have been identified in the literature [7], including multidrug therapy, the presence of gastrointestinal diseases, and drug side effects [7,8,9,10]. New treatments well tolerated by patients are needed to avoid poor adherence and persistence to anti-osteoporotic treatments. However, despite osteopenia is an indicator of low bone density, which increase the risk for fractures, but is not osteoporosis, not drugs exist specifically for it.

There is significant evidence showing the effectiveness of functional foods in preventing bone loss, which has recently led to their increased prophylactic use, especially in postmenopausal women [11,12]. 

Milk proteins (MPs) are important components of the human diet, as they are a major source of nitrogen and essential amino acids. MPs are made up of major sources of protein: casein and whey protein (WP) [13]. In comparison with casein, WP has branched-chain amino acids (BCAAs) [14] and several bioactive proteins with various nutritional and health benefits, such as β-lactoglobulin, α-lactalbumin, and lactoferrin [15,16]. MPs and WPs have already been demonstrated to have positive effects on bone metabolism [17,18,19]. Moreover, clinical studies investigating the role of MP-rich foods in preventing bone loss in postmenopausal women are scarce and contradictory [20,21,22,23]. Considering the need to prevent the progression of osteopenia into osteoporosis, the high rate of non-adherence of OP pharmacological agents, their potential adverse events, and their high cost, further studies are justified to evaluate the effects of functional food against bone loss. 

In our prospective clinical study, we assess the feasibility of a new therapeutic approach for BMD loss prevention in postmenopausal women through the intake of an ice cream enriched with MPs. We also, investigate the effects of WP on osteoblast function and on differentiation and osteoclastogenesis.

## 2. Materials and Methods

### 2.1. Human Study Design

A sample of 62 consecutive postmenopausal women attending the outpatient clinic of the “Mater Domini” Azienda University Hospital in Catanzaro, Italy, were enrolled for this study, to receive 80 g/day of an ice cream enriched with MPs (provided by CALLIPO Gelateria srl Pizzo, Italy) for 3 months (enrolment period between 21 May 2015 and 28 July 2015). This is a feasibility study aimed at investigating certain aspects of the intervention delivery to ensure that it will work in a future, larger clinical study. Furthermore, our aim was to understand to what extent is the intervention is suitable, satisfying, or attractive to study’s recipients. A sample of 21 consecutive postmenopausal women consuming their usual diet and not consuming ice cream enriched with MPs served as the control. Usual diet was defined as a dietary pattern and range of food that participants regularly eat, without calorie restriction or any dietary change. Vanilla-flavored ice cream was provided for this study. The current study design, thus, tested processes related to the intervention arm only [24,25] and our control group did not receive a control ice cream. The protocol was approved by the local ethics committee at the “Mater Domini” University Hospital in Catanzaro, Italy (projects codes 117/2015/CE, approved 14 May 2015). We obtained written informed consent from all postmenopausal women included in the study. The investigation conforms to the principles outlined in the Declaration of Helsinki. 

In this study, we enrolled participants in which postmenopausal status was defined as the presence of a serum follicle-stimulating hormone (FSH) level greater than 40 IU/L (if available) or no natural menses for at least 1 year. We enrolled postmenopausal women (i.e., with a serum follicle-stimulating hormone concentration greater than 40 IU/L or no natural menses for at least 12 months). We excluded women with diabetes, cancer, hypercalcemia, and other diseases or medications that affect bone metabolism (such as liver, kidney, thyroid, hematological, and rheumatic diseases; malabsorption syndromes; use of anti-osteoporotic agents; thyroid hormone replacement therapy; aromatase inhibitors; anti-epileptics; and glucocorticoids). 

If patients took vitamin D and/or calcium supplementation at the time of enrolment, they continued on the same dose throughout the duration of the study. Participants’ age, body mass index (BMI: kg/m^2^; dry weight in meters squared), serum biochemical parameters (glucose, transaminases, creatinine, high sensitivity *C*-reactive protein (CRP), cross-linked *C*-terminal telopeptide of type I collagen (s-CTx), bone-specific alkaline phosphatase (BALP)) were collected before and after 3 months of treatment. The presence of hyperlipidemia, hypertension, and previous fractures were recorded at baseline during participants’ medical visits.

Quantitative ultrasound (QUS) was used to measure the speed of sound (meters per second) and broadband ultrasound attenuation (BUA) (decibels per megahertz) of the heel (SaharaTM Clinical Bone Sonometer, Technologic Srl-Hologic, Italia). In participants with previous fractures within the lower limb or lower extremity, only the opposite calcaneus was measured. A T-score was derived from the value of BUA and expressed as the number of SDs from the mean value of a control gender-matched population [26]. The T-scores were reported as the number of standard deviations below the young adult mean (normal, >−1; osteopenia, from −1 to −2.49; osteoporosis, ≤−2.5) [26]. The coefficient of variation (CV%) was 2% for BUA. Calcaneus BMD, T-score, BALP, and s-CTx were measured at baseline and at the end of the study.

#### 2.1.1. Clinical Outcomes and Treatment

The primary outcomes were: (1) change in BMD, which was assessed by QUS on individuals’ right calcaneus [26]; (2) change in key bone turnover markers such as serum BALP and s-CTx. Postmenopausal women in the intervention group received 80 g/day of the ice cream enriched with MPs. Functional ice cream provided a total of 173.6 kcal, 18.7 g carbohydrates, and 8.5 g lipids, respectively (Supplementary Table S1). The final protein content in functional vanilla ice cream was 4.8 g/80 g, while a non-functional vanilla ice cream usually contains 2.64 g/80 g; the WP content was 0.96 g/80 g and the casein content was 3.84 g/80 g [15,16]. It was not possible to increase the WP content in the ice cream due to the difficulty of obtaining a stable emulsion [27].

#### 2.1.2. Participants’ Biochemical Evaluation 

After fasting overnight, venous blood was collected into vacutainer tubes (Becton and Dickinson, Plymouth, England) and centrifuged within 4 h. Serum glucose, creatinine, total cholesterol (TC), high density lipoprotein cholesterol (HDL-C), triglycerides (TG), CRP, and transaminases were measured by chemiluminescent immunoassay on COBAS 8000 (Roche, Switzerland), according to the manufacturer’s instructions. BALP and s-CTx were assessed by chemiluminescent immunoassay on Liaison^®^ XL (DiaSorin, Saluggia, Italy), according to the manufacturer’s instructions. Quality control was assessed daily for all determinations.

### 2.2. In Vitro Study

#### 2.2.1. Chemicals, Reagents, and Materials

WP isolated and ultra-centrifugated (97% protein), dexamethasone (DEX), McCoy’s 5A modified medium, fetal bovine serum (FBS), and trypsin-EDTA were purchased from Gibco (Life Technologies, Grand Island, NY, USA), while penicillin/streptomycin was purchased from Lonza (Basel, Switzerland). Methanol and all other solvents and reagents were of analytical grade (Carlo Erba, Milan, Italy).

#### 2.2.2. Cell Culture

The human osteoblast-like cell line Saos-2 was obtained from American Type Culture Collection ATCC (Italy Office, via Venezia 23, 20,099 Sesto San Giovanni, Milan, Italy). The cells were maintained in McCoy’s 5A (Gibco, Carlsbad, CA, USA) supplemented with 15% fetal bovine serum (Gibco, Carlsbad, CA, USA) and 1% penicillin streptomycin (PAA, Linz, Austria), at 37 °C in 5% CO2, then harvested by trypsinization and subcultured twice weekly. In all the experiments, Saos-2 cells were incubated with dexamethasone 10 nM in order to obtain a more differentiated cell line. 

#### 2.2.3. Cell Proliferation

Saos-2 cells were seeded in 6-well dishes with a density of 2 × 10^5^ cells/well. Cells were grown in serum-free medium and incubated with WP 0.5; 1; 2 mg/mL for 24 h. The exact cellular number was determined by cell count through an optical microscope. Moreover, Saos-2 cells were treated with a high dose of 2 mg/mL of WP and 40 μM of ERK1/2 inhibitor PD184352 (#PZ0181, Sigma Aldrich, St. Louis, MO, USA) [28].

#### 2.2.4. Western Blotting 

Saos-2 cells were seeded in 6-well dishes with a density of 2 × 10^5^ cells/well. Cells were grown in serum-free medium and incubated with WP 0.5; 1; 2 mg/mL for 10 min. Cells were lysed in Mammalian Protein Extraction Reagent (M-PER) (Pierce, Thermo Fisher Scientific, Waltham, MA, USA). Western blot analyses of proteins from cell lysates were performed according to standard procedures. The following antibodies were used: rabbit anti-p Extracellular Signal-regulated Kinase (ERK)1/2 (9101), rabbit anti-pAkt (9271S), and mouse anti-β-actin (3700). 

#### 2.2.5. ALP Activity

Saos-2 cells were seeded with a density of 2 × 10^5^ cells/well in 6-well dishes. Cells were incubated with WP 0.5; 1; 2 mg/mL for 24 h. Cells were lysed with Mammalian Protein Extraction Reagent (M-PER) (Pierce, Thermo Fisher Scientific). Protein concentration was determined using Bradford assay and ALP activity was determined by the p-nitrophenyl phosphate (pNPP) colorimetric method (WAKO Chemicals USA, Richmond, VA, USA).

#### 2.2.6. Real Time-PCR

Saos-2 cells were seeded at a density of 1 × 10^6^ cells/well in 100 mm culture dishes. Cells were grown in serum-free medium and incubated with WP 0.5; 1; 2 mg/mL for 24 h. Total RNA from cells were extracted with Trizol reagent (Life Technologies, Cramlington, UK) according to the manufacturer’s instructions. cDNA was synthesized from 1 µg total RNA, using a High-Capacity cDNA Reverse Transcription Kit (Applied Biosystems, Foster City, CA, USA). mRNA expression of RANKL, OPG, RUNX2, COL1A, and β-ACTIN were quantified by real time-PCR using SYBR^®^ Green dye (SYBR^®^ Green PCR Master Mix, Applied Biosystems, Foster City, CA, USA) (Supplementary Table S2). Moreover, Saos-2 cells were treated with a high dose of 2 mg/mL of WP and 40 μM of MEK/ERK1/2 inhibitor PD184352 (#PZ0181, Sigma Aldrich, St. Louis, MO, USA) [28].

### 2.3. Statistics

Data are reported as mean ± standard deviation (SD). For the clinical study, a Χ^2^ test was used to test prevalence difference between groups and an independent unpaired-samples t-test was performed to compare the difference between means. We calculated and compared the means of changes in clinical outcomes (such as T-score, BMD, BALP, and S-CTx) between treatment groups.

A paired Student’s *t*-test (two-tailed) was used to assess changes in the clinical parameters from baseline to follow-up (within-group variation). The General Linear Model was used to adjust the T-score, BMD, and BALP changes for potential confounders (such as BMI and vitamin D supplementation). Adherence to the treatment was evaluated by an interview. All comparisons were performed using SPSS 25.0 for Windows (IBM Corporation, New York, NY, USA). In both studies, significant differences were assumed to be present at *p* < 0.05 (two-tailed).

In relation to the in vitro study, data resulted from a mean of at least three independent experiments and were analyzed with GraphPad Prism 5.0 software using a two-tailed Student’s *t*-test or Linear Regression trend.

## 3. Results

### 3.1. Human Study

#### 3.1.1. Clinical Characteristics of Participants

The mean age of the enrolled population was 63.9 ± 7 years. The mean BMD at baseline was 0.380 ± 0.08 g/cm^2^ and mean T-score was −1.86 ± 0.74 SD. Table 1 shows the clinical characteristics of participants according to treatment. We found a significant difference of BMI value between the two groups (*p* = 0.026). No other variables were significantly different between groups at baseline.

#### 3.1.2. Clinical Characteristics Changes at Follow-Up and Outcomes of the Study

The participants in the MPs’ ice-cream group had a high adherence to the protocol (i.e., >80% of the prescribed treatment). All the enrolled patients reported that the ice cream had a good taste, consistency, and palatability. No adverse events were reported during 3 months of MPs’ ice-cream intervention in postmenopausal women.

The changes in the clinical outcomes after treatments are shown in Table 2. After 3 months, we found a greater reduction in BALP in the MPs’ ice-cream group than the control group, respectively (−2.73 ± 4.4 ug/L vs. −0.62 ± 2.9 ug/L, *p* = 0.038; Table 2). The percentage of BALP reduction was −13.8% in the MPs’ ice-cream group and 1.04% in the control group (*p* = 0.021; Table 2). At the end of the study, the percentage change of the BMD was −2.2% in the MPs’ ice-cream group and −8.3% in the control group (*p* = 0.029). These results were also confirmed after adjustment for BMI and vitamin D supplementation. Indeed, the adjusted BMD change was −0.01 ± 0.01 g/cm^2^ and −0.04 ± 0.01 g/cm^2^ in the MPs’ ice-cream and the control group, respectively (*p* = 0.032; Table 2). The adjusted BALP change was −2.86 ± 0.7 ug/L and −0.38 ± 0.9 ug/L in the MPs’ ice-cream and the control group, respectively (*p* = 0.040; Table 2). We also found a significant difference of T-score value between groups (*p* = 0.026; Table 2). No other variables were significantly different between groups at follow-up visit.

**Figure 1:**
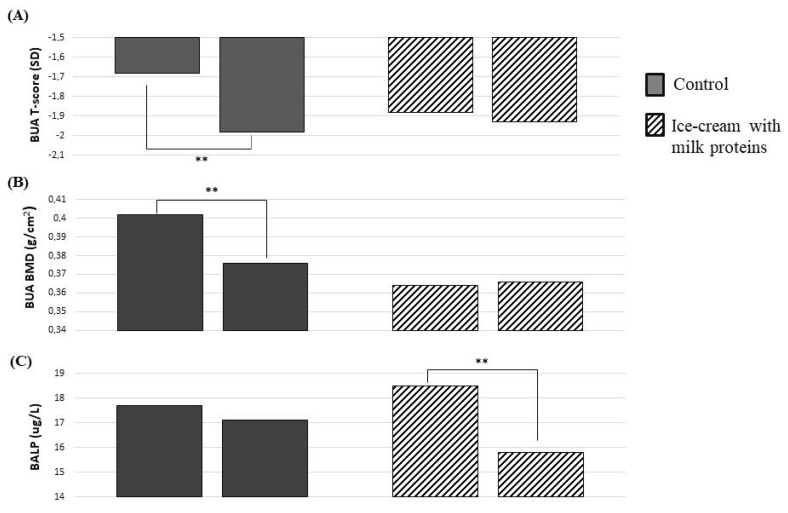
Changes in bone parameters after 3 months with or without milk protein-enriched ice cream. T-Score (**A**), BMD (**B**), and BAP (**C**). Abbreviations: BMD = bone mineral density; BALP = bone alkaline phosphatase. Paired Student’s *t*-test: ** *p* < 0.01.

In the MPs’ ice-cream group, we did not detect a significant loss of BMD in comparison to their baseline value (0.376 ± 0.08 to 0.367 ± 0.09 g/cm^2^, *p* = 0.14; Figure 1B). On the contrary, women in the control group had a loss of BMD compared with their baseline value (0.402 ± 0.09 to 0.365 ± 0.06 g/cm^2^, *p* = 0.002; Figure 1B) after 3 months. The T-score also had a similar reduction for BMD among the two groups (Figure 1A). A reduction of BALP was detected only in the MPs’ ice-cream group in comparison to the baseline (in the MPs’ ice-cream group, from 18.5 ± 8 to 15.8 ± 7 ug/L, *p* = 0.001; in the control group, from 17.7 ± 9 to 17.1 ± 7 ug/L, *p* = 0.37; Figure 1C).

### 3.2. In Vitro Study

#### 3.2.1. Whey Proteins Induces pERK1/2 and pAKT Protein Expression Level on Saos-2

**Figure 2:**
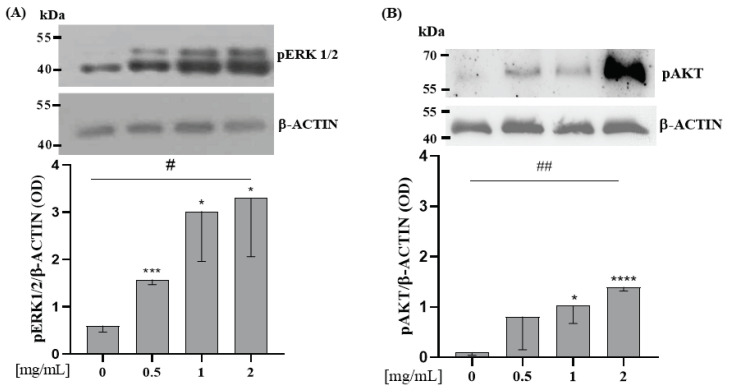
WP induces pERK 1/2 and pAKT protein expression level on Saos-2 cells. Semi-confluent cultures of human osteoblast-like cells (Saos-2) were incubated with WP 0.5, 1, and 2 mg/mL in serum-free medium for 10 min. Cell proteins were analyzed by Western blotting with antibodies specific to phosphorylated ERK1/2 (**A**), AKT (**B**), and β-ACTIN. Data are represented as mean ± SD. Statistical analysis: Student’s *t*-test vs. 0: * *p* < 0.05, *** *p* < 0.001, **** *p* < 0.0001 (Student’s *t*-test); # *p* < 0.05, ## *p* < 0.01 (Test for linear trend).

Saos-2 cells were incubated with WP 0.5; 1; 2 mg/mL for 10 min. All these doses of WP increased pERK1/2 expression compared to the control (0 vs. 0.5 mg/mL WP, *p* < 0.001; 0 vs. 1 mg/mL WP, *p* = 0.017; 0 vs. 2 mg/mL WP, *p* = 0.019) (Figure 2A). Figure 2B shows that only 1 and 2 mg/mL of WP increased pAKT. Moreover, WP incubation increased the protein expression levels of pERK1/2 and pAKT in a dose-dependent manner in comparison to the control (Linear Regression: *p* = 0.002 and *p* = 0.016, respectively; Figure 2A,B).

#### 3.2.2. Whey Proteins Increase mRNA Expression Levels of RUNX2 and Decrease RANKL/OPG Ratio mRNA Expression Levels on Saos-2

**Figure 3:**
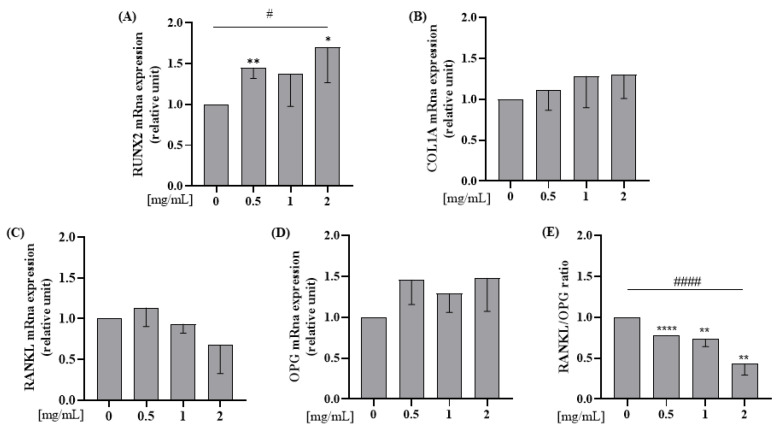
WP increases mRNA expression levels of RUNX2 and decreases RANKL/OPG ratio mRna expression levels on Saos-2 cells. Semi-confluent cultures of human osteoblast-like cells (Saos-2) were incubated with WP 0.5,1 and 2 mg/mL for 24 h. Then, mRNA expression levels of (**A**) RUNX2, (**B**) COL1A, (**C**) RANKL, and (**D**) OPG were measured by RT-PCR. Data were analyzed using the 2−ΔΔCq method and normalized to β-actin. Data are represented as mean ± SD. Statistical analysis: Student’s *t*-test vs. 0: * *p* < 0.05, ** *p* < 0.01 ****, *p* < 0.0001 (Student’s *t*-test); # *p* < 0.05, #### *p* < 0.0001 (Test for linear trend). Abbreviations: RUNX2 = runt-related transcription factor 2; COL1A = type 1 collagen; RANKL = receptor activator of nuclear factor kappa-Β ligand; RANK = receptor activator of nuclear factor kB; OPG = osteoprotegerin.

Saos-2 cells were incubated with WP 0.5; 1; 2 mg/mL doses for 24 h. RUNX2 mRNA expression level was significantly increased by 0.5 and 2 mg/mL of WP in comparison to the control (Student’s *t*-test: *p* = 0.004 and *p* = 0.048, respectively; Figure 3A). Furthermore, all doses of WP increased RUNX2 in a dose-dependent manner in comparison to the control (Test for linear trend: *p* = 0.031; Figure 3A). WP had no effect on RANKL and OPG mRNA expression when each marker was considered individually (Figure 3C,D) but, WPs decreased RANKL/OPG ratio mRNA expression levels, in a dose-dependent manner, compared to the control (0 vs. 0.5 mg/mL WP, *p* < 0.001; 0 vs. 1 mg/mL WP, *p* = 0.007; 0 vs. 2 mg/mL WP, *p* = 0.002; Test for linear trend: *p* < 0.001; Figure 3E). 

#### 3.2.3. Whey Proteins Increase Osteoblast Proliferation and ALP Enzymatic Activity on Saos-2 Cell Line

**Figure 4:**
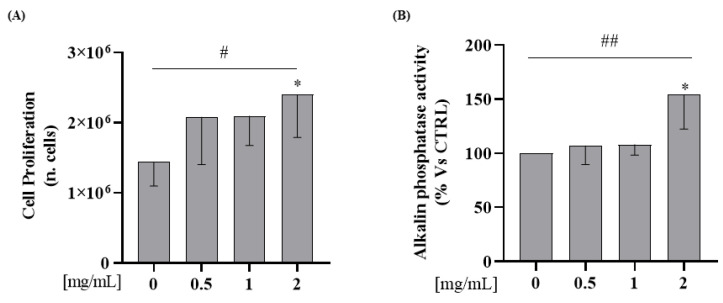
WP increases cellular proliferation and alkaline phosphatase activities on Saos-2 cells. Semi-confluent cultures of human osteoblast-like cells (Saos-2) were incubated with WP 0.5,1 and 2 mg/mL for 24 h. (**A**) Cell proliferation was determined by counting the number of cells in each well. (**B**) ALP activity was measured by pNPP method. Data are represented as mean ± SD. Statistical analysis: Student’s *t*-test vs. 0: * *p* < 0.05 (Student’s *t*-test); # *p* < 0.05, ## *p* < 0.01 (Test for linear trend).

To test the hypothesis that WPs increase osteoblast proliferation, Saos-2 cells were incubated with WP 0.5; 1; 2 mg/mL for 24 h. As shown in Figure 4A, incubation with 2 mg/mL of WP increases cellular proliferation in comparison to the control (Student’s *t*-test: *p* = 0.03). Furthermore, we observed an increase of proliferation in a dose-dependent manner in comparison to the control (Test for linear trend: *p* = 0.03; Figure 4A).

In order to investigate WPs’ capability to affect osteoblasts differentiation, ALP activity was determined on cell lysate by using *p*-Nitrophenyl Phosphate (pNPP). We observed, in Saos-2 cells, an increase of ALP activity levels at a 2 mg/mL dose in comparison to the control (Student’s *t*-test: *p* = 0.04). In addition, WP incubation increased the protein expression levels of ALP activity in a dose-dependent manner compared to the control (Test for linear trend: *p* = 0.02; Figure 4B).

#### 3.2.4. Involvement of ERK1/2 on WP-Induced Differentiation and Proliferation in Saos-2 Cells

We next evaluated the involvement of extracellular signal-regulated protein kinase (ERK1/2), a member of the mitogen-activated protein kinase family, in WP-induced activation of the differentiation and proliferation process. We confirm the role of WP on ERK1/2 by silencing its expression using PD184352, a highly selective non-competitive inhibitor of MEK/ERK1/2, and assessed its effects on RUNX2 and ALP mRNA expression and cells proliferation in vitro.

**Figure 5:**
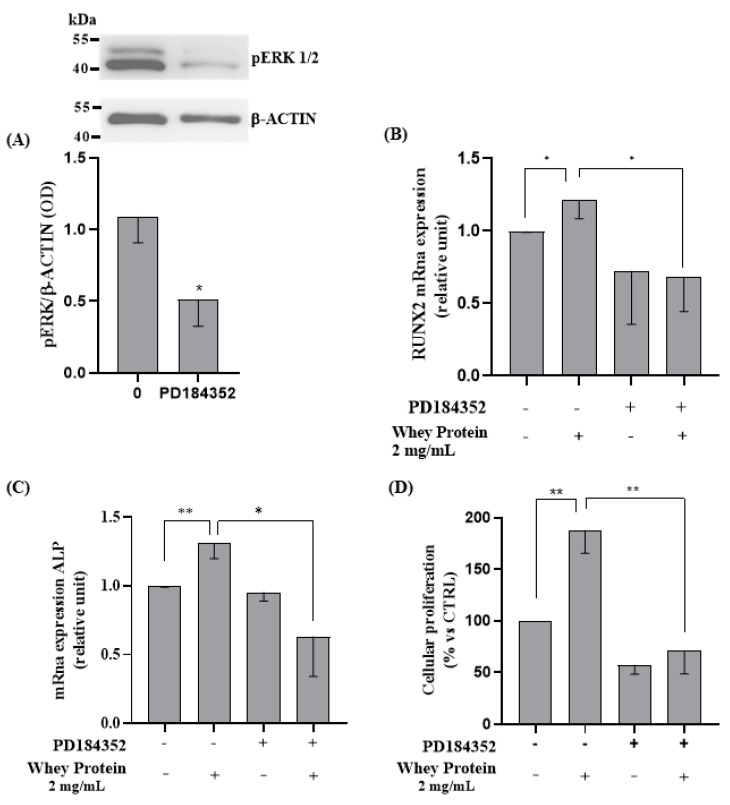
ERK1/2 inhibition role in WP-induced proliferation and differentiation on Saos-2 cells. Semi-confluent cultures of human osteoblast-like cells (Saos-2) were pretreated with PD184352 for 1 h, then incubated with 2 mg/mL of WP for 24 h. Cell proteins were analyzed by Western blotting, with antibodies specific to pERK1/2 and β-actin (**A**). Then, mRNA expression levels of (**B**) RUNX2 and (**C**) ALP were measured by RT-PCR. Data were analyzed using the 2−ΔΔCq method and normalized to β-actin. Cell proliferation (**D**) was determined by counting the number of cells in each well. Data are represented as mean ± SD. Statistical analysis: * *p* < 0.05, ** *p* < 0.01 (Student’s *t*-test). Abbreviations: RUNX2 = runt-related transcription factor 2; PD184352 = MEK/ERK inhibitor; ALP = alkaline phosphatase.

PD184352 effectively suppressed ERK1/2 protein levels (Student’s *t*-test: *p* = 0.017, Figure 5A). As expected, inhibition of ERK1/2 by PD184253, in the presence of 2 mg/mL of WP, reverted the effects on both the RUNX2 and ALP mRNA expression and cells proliferation, compared to cells treated with only 2 mg/mL of WP (Student’s *t*-test: *p* = 0.028, *p*= 0.004, and *p* = 0.003, respectively; Figure 5B–D).

## 4. Discussion

An important result of the present feasibility work is that WPs added to ice cream are palatable and represent a good way to deliver crucial branched amino acids to osteopenic postmenopausal women. This study was conducted on women after menopause and investigated the effects of the assumption of a functional food on bone loss. The intervention consisted of the assumption of 4.8 g/80 g (WP 20%) of MPs for 3 months compared to the participant’s usual diet. 

The main result of this study is that the consumption of MP-enriched ice cream could protect against bone loss in postmenopausal women, potentially due to ERK1/2 activation. In addition, those women who consumed the MP-enriched ice cream reduced their BALP to a greater extent than those who did not consume the functional food. In particular, we demonstrated that postmenopausal women who consumed MP-enriched ice cream prevented physiological loss of BMD, and significantly reduced their BALP concentrations by 13.1% in a 3-month period. It is recognized that feasibility studies do not evaluate the outcome of interest, but they are used to estimate important parameters which are needed to design the main study. Therefore, our results must be interpreted with caution as they only generate hypotheses for future studies. 

BALP is an important biomarker of bone formation produced by osteoblasts [29]. Different studies described the BALP increase in postmenopausal women with high bone turnover [29]. As a consequence, BALP serum reduction is observed following the anti-resorptive therapy [30,31,32]. Interestingly, the percentage reduction in BALP after treatment with MP-enriched ice cream was similar to that obtained after treatment with anti-osteoporotic drugs [32].

In one study, BALP was significantly decreased in response to both alendronate 10 mg/day and cyclical etidronate after 3 months (of ~ 10%, as in our study) [32]. In line with our findings, the reduction of serum BALP was shown even after 3 months of consumption of functional food in osteoporotic women [24,25,33]. We also observed that BMD was preserved in patients who consumed the MP-enriched ice cream compared to the control group. Some studies also described bone mass preservation and osteoporosis risk attenuation thanks to suitable dietary interventions [25], while a systematic review suggests that protein intake may play a beneficial role in the maintenance and loss of BMD in the elderly [34]. In particular, studies suggested that WP supplementation and milk basic protein might have a positive impact on BMD [35]. Moreover, an in vivo study also revealed that lactoferrin, a component of WP, helps to prevent BMD loss in ovariectomized rats [36]. Thus, we believe that our finding may have important clinical implications, and that this study represents a necessary first step in exploring novel applications of MP-enriched ice cream in future larger-scale studies.

The results obtained in the clinical study prompted us to test the efficacy of WP in vitro. This was also considering that they represent 20% of MPs [16] and they contain different bioactive proteins, including β-lactoglobulin, α-lactalbumin, and lactoferrin [15,16]. We used the Saos-2 cell line, which represents a valid model for the study of events associated with the late stage of osteoblastic differentiation in human cells, in particular in the presence of dexamethasone [37,38]. 

We showed that WP: (i) increased the protein expression of ERK1/2 and AKT pathways; (ii) increased the expression of RUNX2, an important transcription factor for differentiation and bone formation; (iii) increased the enzymatic activity of ALP as well as cell proliferation; (iv) reduced the RANKL/OPG ratio, suggesting a potential role in bone resorption. Finally, we showed how WPs exert their effects on bone cells through the ERK1/2 pathways.

First of all, we evaluated the effects of WP on the ERK1/2 e AKT pathways (Figure 2), which promote osteoblast proliferation and differentiation [39,40]. No studies have previously shown similar effects on bone cells. This effect may be attributable to the presence of lactoferrin in the WP [41,42]. Activation of the ERK1/2 pathway is known to promote the expression of osteogenic genes such as RUNX2 and ALP, and enhance the proliferative activity of osteoblasts [43].

In line with the literature, our study showed that WP increased osteoblast proliferation and differentiation markers. In detail, we demonstrated that WP increased cell proliferation and alkaline phosphatase activity after 24 h of treatment. Similarly, a study of Xu et al. carried out in rat osteoblasts showed that WPs exposure to the culture medium stimulate cell growth and ALP production in a dose-dependent manner [44]. 

The RUNX2 protein is a potent osteoblast transcription factor which promotes the expression of ALP in the early differentiation phase [45,46], and it has been reported that WPs increase the expression of RUNX2 in Saos-2 [17]. These reports are in agreement with our findings, where increased RUNX2 expression supported bone differentiation.

We also measured the mRNA expression of cytokines related to bone metabolism, namely RANKL and OPG. RANKL is secreted by osteoblasts following binding to RANK on the surface of pre-osteoclasts; RANKL thus stimulates the activation and development of osteoclasts. Since the RANKL/OPG ratio was reduced in our study, the balance between formation and resorption seems in favor of formation [47,48]. 

With the maximal dose of WP (2 mg/mL) over 24 h of treatment, the number of osteoblasts doubled vs. no treated cells. However, based on our results, an anabolic effect of whey proteins cannot be fully confirmed. We hypothesize that whey proteins affect bone turnover at an early stage, i.e., on osteoblasts and when pre-osteoclasts link RANKL. An effect of whey protein in inhibiting bone resorption cannot be excluded, but it seems a late phenomenon with the involvement of osteoclasts subsequent to osteoblasts. Further in vitro studied are needed to better clarify these mechanisms. 

Our results showed that the high-dose group always had a higher OPG mRNA expression and a lower RANKL expression, albeit insignificant. However, the RANKL/OPG ratio showed a reduction compared to the control group, suggesting that WP may have an effect on osteoclastogenesis. 

In line with our results, lactoferrin treatment significantly suppressed the RANKL/OPG mRNA ratio in ovariectomized rats [36,49].

To further characterize the molecular mechanisms that regulate WP in osteoporosis, we examined the contribution of the ERK1/2 cell signaling pathway in cultured osteoblasts. To prove ERK1/2 was the mediator of the observed beneficial effect, we specifically inhibited ERK1/2 by using PD184352 and observed an inhibition of cell proliferation and differentiation. This inhibition was mediated by the suppression of transcription factor gene expression RUNX2 and the ALP activity. Taking all this together, WPs activate the ERK1/2 pathway, which in turn promotes the expression of osteogenic genes such as RUNX2 and ALP, and enhances the proliferative activity of osteoblasts. Conversely, inhibition of this pathway, in the presence of WPs, suppresses the expression of these genes and cell proliferation. These in vitro results corroborate the effects of MP-enriched ice cream on BMD and BALP in postmenopausal women. In addition, we have developed and tested an enriched food which is more patient-accepted than powder and tablet formulations [50]. In addition, ice cream also contains calcium, a constituent mineral of bone [51].

Our study has some limitations. First, our study was not double blind and randomized. The study did not include a group with ice cream without MPs. Our clinical study did not exclude possible effects by other components of the milk proteins, but our in vitro study aimed specifically at investigating the effects of whey protein alone. WPs have been shown to have a fast rate of digestion and absorption, producing a rapid peak in plasma amino acids compared with casein [52]. Whey also has a higher branched-chain amino acids content compared with casein. For these reasons, we focused our experiments only on WP. However, an effect of casein or other components of MPs cannot be excluded.

In addition, we have not evaluated eating habits; therefore, the presence of other dietary factors that may have influenced our findings cannot be excluded. However, our in vitro study is consistent with a beneficial role of MP. Although it has been demonstrated that QUS densitometry can detect short-term changes in BMD [25,53,54], it is necessary to design future long-term studies involving the use of DXA (the reference technology for bone mass study) to confirm the effects of a functional ice cream on bone.

For this reason, the findings obtained must be treated with caution and should only serve to generate new hypotheses for future clinical investigations. Despite BMI was different between groups at baseline, body weight chance was not significantly different between groups at follow-up. However, it has been demonstrated that the BALP value is not associated with BMI [55].

In our research, WPs increased ALP expression and activity in vitro, while MP-enriched ice cream decreased BALP in humans. This finding may seem controversial, but it is not. Moreover, some studies on anti-osteoporosis agents showed a BALP reduction after pharmacological treatment, and this decrease in BALP was associated with long-term increases in bone mass [56,57].

Finally, we also did not evaluate the effects of WPs on osteoclast cell lines. To date, there is no cellular model exactly miming postmenopausal osteoporosis. However, we used a Saos-2 cell line that represents a useful model to identify targets and test new medications for osteoporosis treatment [58].

Despite these limitations, due to their potent proliferative actions on osteoblast, our findings could suggest that MPs, and specifically WPs, have anabolic effects on bone. Since amino acids derived from dietary proteins are essential for the synthesis of new bone matrix, a restriction of the protein diet adversely affects bone health [59].

In relation to these observations, a possible synergistic action on the bone metabolism of the various proteins contained in the WPs is hypothesized. However, further studies are needed to fully understand the mechanisms underlying the beneficial effect for humans of consuming foods containing WPs.

## 5. Conclusions

Our clinical study seems to suggest that a functional ice cream containing MPs may modulate markers of bone metabolism in postmenopausal women with osteopenia.

Despite this clinical study, we cannot exclude a potential effect by other components of MPs different from WP, with the in vitro study strongly supporting a role for WP.

The observed results, both in vitro and in humans, may be due to the high nutritional value of MPs as well as the presence of various bioactive compounds. In conclusion, a functional food with MPs or WPs could have a physiological role in bone growth as well as a potential therapeutic role in osteoporosis without causing adverse events. However, larger clinical studies are needed to confirm our findings, along with other in vitro investigations testing other molecules containing MPs.

## Figures and Tables

**Table 1:** Baseline demographic and clinical characteristics of participants according to intervention.

Variables	Control (*n* = 21)	MPs Ice Cream (*n* = 41)	*p*-Values
Age (years)	66 ± 6	63 ± 7	0.11
Menopausal status (years)	15 ± 7	17 ± 9	0.37
Weight (kg)	67 ± 11	62 ± 9	0.14
BMI (kg/m^2^)	28.3 ± 5	25.7 ± 3	0.026
BUA T-score (SD)	−1.75 ± 0.7	−1.91 ± 0.8	0.42
BUA BMD (g/cm^2^)	0.394 ± 0.09	0.373 ± 0.08	0.37
Glucose (mg/dL)	92 ± 10	89 ± 8	0.24
Creatinine (mg/dL)	0.71 ± 0.09	0.71 ± 0.09	0.97
TC (mg/dL)	224 ± 32	208 ± 47	0.13
HDL-C (mg/dL)	70 ± 17	64 ± 19	0.19
TG (mg/dL)	93 ± 29	102 ± 41	0.30
AST (IU/L)	20 ± 5	20 ± 7	0.70
ALT (IU/L)	18 ± 9	19 ± 10	0.91
CRP (mg/L)	3.6 ± 1.2	3.2 ± 0.6	0.17
S-CTX (ng/mL)	0.58 ± 0.31	0.55 ± 0.28	0.68
BALP (ug/L)	18.1 ± 8	18.9 ± 8	0.68
*Prevalence*			
Smoking habit (%)	24	12	0.28
Hyperlipidemia (%)	52	61	0.59
Hypertension (%)	33	46	0.41
Total fractures (%)	38	46	0.59
*Medication*			
Calcium (%)	10	15	0.70
Vitamin D (%)	43	29	0.39

Note: BMI = body mass index; BUA = broadband ultrasound attenuation; BMD = bone mineral density; TC = total cholesterol; HDL-C = high density lipoprotein cholesterol; TG = triglycerides; AST = aspartate aminotransferase; ALT = alanine aminotransferase; CRP = c-reactive protein; S-CTX = serum carboxyterminal crosslinked telopeptide of type I collagen; BALP = bone alkaline phosphatase.

**Table 2:** Changes in participants’ characteristics at follow-up according to intervention.

Variables	Control (*n* = 19)	MPs’ Ice Cream (*n* = 36)	*p*-Values
Weight (kg)	−0.78 ± 1.2	−0.63 ± 2.2	0.74
BMI (kg/m^2^)	−0.42 ± 0.6	−0.15 ± 0.7	0.13
BUA T-score (SD)	−0.30 ± 0.39	−0.05 ± 0.36	0.026
^a^BUA T-score (SD)	−0.32 ± 0.09	−0.04 ± 0.06	0.020
BUA BMD (g/cm^2^)	−0.04 ± 0.04	−0.01 ± 0.04	0.029
^a^BUA BMD (g/cm^2^)	−0.04 ± 0.01	−0.01 ± 0.01	0.032
Glucose (mg/dL)	−0.53 ± 5.8	−1.47 ± 6.5	0.58
Creatinine (mg/dL)	−0.01 ± 0.05	−0.01 ± 0.08	0.86
CRP (mg/L)	−0.15 ± 0.9	0.10 ± 0.6	0.38
S-CTX (ng/mL)	0.01 ± 0.14	−0.02 ± 0.12	0.46
BALP (ug/L)	−0.62 ± 2.9	−2.73 ± 4.4	0.038
^a^ BALP (ug/L)	−0.38 ± 0.9	−2.86 ± 0.7	0.046
BALP (%)	−0.21 ± 16	−13.1 ± 22	0.016
^a^ BALP (%)	1.04 ± 5	−13.8 ± 3	0.021

Note: ^a^ Adjusted for BMI and Vitamin D supplementation. BMI = body mass index; BUA = broadband ultrasound attenuation; BMD = bone mineral density; S-CTX = serum carboxyterminal crosslinked telopeptide of type I collagen; BALP = bone alkaline phosphatase; CRP = c-reactive protein.

## Data Availability

Not applicable.

## Supplementary Materials

The following supporting information can be downloaded at: https://www.mdpi.com/article/10.3390/nu15020344/s1, Table S1: Nutrient composition of vanilla ice cream enriched with milk proteins; Table S2: Real-time primer sequences.
