# Supplementary material for: Effects of a Functional Ice Cream Enriched with Milk Proteins on Bone Metabolism: A Feasibility Clinical Study and In Vitro Investigation
Source: Nutrients. 2023 Jan 10;15(2):344. doi: 10.3390/nu15020344 (PMC9865372; doi:10.3390/nu15020344)
Supplement: Supplementary file 1 [file nutrients-15-00344-s001.zip › nutrients-2079541-supplementary.pdf]

Supplemental Table S1. **Nutrient composition of vanilla ice-cream enriched with Milk Proteins.**

**Nutrients/100 g**	**Value**
Energy (Kcal)	217
Proteins (g)	6.03
Total carbohydrates (g)	23.40
Glucose (g)	0.75
Fructose (g)	0.67
Lactose (g)	3.65
Sucrose (g)	16.28
Maltose (g)	1.19
Total fiber (g)	2.20
Total Lipids (g)	10.56
SFA (%)	72.72
MUFA (%)	25.69
PUFA (%)	1.52
Myristic acid (C 14:0) (%)	11.86
Palmitic acid (C 16:0) (%)	33.06
Stearic acid (C 18:0) (%)	11.33
Oleic acid (C 18:1) (%)	22.97
Sodium (mg/kg)	649

Note. SFA = saturated fatty acids; MUFA = monounsaturated fatty acids; PUFA = polyunsaturated fatty acids.

***Supplemental Table S2. Real*-Time primer sequences.**

**Gene**	**Forward**	**Reverse**
**RUNX2**	5’-TTACTTACACCCCGCCAGTC -3’	5’-TATGGAGTGCTGCTGGTCTG-3’
**ALP**	5’-GACCTTGACCCCCACAAT-3’	5’-GCTCTACTGCATTCCCCTC-3’
**COL1A**	5’-CCCCAGCCCACAAAGAGTCTA -3’	5’-CTGTACGCAGGTGATTGGTG-3’
**RANKL**	5’-AGAGCGCAGATGGATCCTAA-3’	5’-TTCCTTTTGCACAGCTCCTT-3’
**Osteoprotegerin**	5’-TGCAGTACGTCAAGCAGGAG-3’	5’-GTGTCTTGGTCGCCATTTTT-3’
**β-ACTIN**	5’-GACTGTGACGAGTTGGCTGA-3’	5’-CTGGAGAGGAGCAGAACTGG-3’
